# Draft Genome Sequence of the Fish Pathogen *Piscirickettsia salmonis*

**DOI:** 10.1128/genomeA.00926-13

**Published:** 2013-11-07

**Authors:** Mark Eppinger, Katelyn McNair, Xhavit Zogaj, Elizabeth A. Dinsdale, Robert A. Edwards, Karl E. Klose

**Affiliations:** South Texas Center for Emerging Infectious Diseases, University of Texas at San Antonio, San Antonio, Texas, USAa; Computational Science Research Center, San Diego State University, San Diego, California, USAb; Department of Biology, San Diego State University, San Diego, California, USAc

## Abstract

*Piscirickettsia salmonis* is a Gram-negative intracellular fish pathogen that has a significant impact on the salmon industry. Here, we report the genome sequence of *P. salmonis* strain LF-89. This is the first draft genome sequence of *P. salmonis*, and it reveals interesting attributes, including flagellar genes, despite this bacterium being considered nonmotile.

## GENOME ANNOUNCEMENT

*Piscirickettsia salmonis* causes a systemic disease in salmonid fish, targeting the kidneys, liver, spleen, intestines, brain, ovaries, and gills (1, 2). The disease is primarily associated with fish found in seawater. Transmission between fish occurs at a high rate in farmed salmon, causing economic losses to the salmon industry. *P. salmonis* is a facultative intracellular pathogen that is able to replicate in a number of fish cell lines. The bacteria can induce vacuolation and apoptosis in infected cells, which leads to detachment and death (3–5). *P. salmonis* forms biofilms under stress conditions, and this may be a means of its interepidemic persistence (6). The genetic basis of *P. salmonis* pathogenesis and environmental persistence is poorly understood. Here, we report the 3,388,517-Mbp (G+C content, 39.2%) draft genome sequence of *P. salmonis* strain LF-89, procured in Chile in 1989, which represents the first reported isolate of this bacterium (1).

Genomic DNA was subjected to next-generation Illumina MiSeq (300-bp insert size, 100-bp paired-end reads) and 454 FLX XLR (3-kb insert size) hybrid sequencing followed by assembly as previously described (7). The individual and hybrid assemblies were generated using the Celera and Velvet assemblers, respectively (8, 9). The Institute for Genome Sciences (IGS) Annotation Engine and Manatee were used for structural and functional annotation and visualization of the 2,514 contigs (10).

Contributing to the difficulty in assembling the *P. salmonis* genome is the presence of two different active transposons: a single transposase gene flanked by a pair of 28-bp indirect repeats, which appears in 1.5% of the sequencing reads, and a single transposase gene that is flanked by 288-bp direct repeats. Interestingly, one copy of the 288-bp repeat can be found in 10% of the reads, whereas the corresponding transposase gene is only present in 1.4% of the reads, suggesting that the 288-bp repeat is present throughout the genome as a single repeated sequence.

Notable aspects to the *P. salmonis* genome sequence include the presence of type IV pilus genes, which may represent the appendages seen as the bacteria come into contact with fish cells (11), and bacteriophage genes that may represent the phage particles associated with *P. salmonis* (12). Type IV secretion system/conjugation genes are present in large clusters, which may be critical for intracellular survival and/or replication. Finally, the presence of flagellar and chemotaxis genes suggests that *P. salmonis* may be capable of flagellum-mediated motility, despite being characterized as nonmotile. The *P. salmonis* flagellar gene organization is almost identical to that of *Vibrio cholerae* (13), and the presence of FlhF and FlhG homologues (14) suggests that *P. salmonis* synthesizes a single polar flagellum.

This genome sequence will facilitate comprehensive bioinformatic and phylogenetic analyses, thus expanding our understanding of the pathogenesis of *P. salmonis*. These data should prove useful for the development of diagnostic and preventive tools in order to enhance the salmon farming industry and prevent future economic losses.

### Nucleotide sequence accession numbers.

The genome sequence has been deposited in DDBJ/EMBL/GenBank under the accession no. ASSK00000000. The version described in this paper is version ASSK02000000.
